# CircZNF367 promotes osteoclast differentiation and osteoporosis by interacting with FUS to maintain CRY2 mRNA stability

**DOI:** 10.1186/s13018-023-03955-7

**Published:** 2023-07-11

**Authors:** Mingsi Deng, Zhengguang Wang, Jia Luo, Heng Cao, Yong Li, Liangjian Chen, Gengyan Liu

**Affiliations:** 1 Department of Orthopedics, The Third Xiangya Hospital, Central South University, No.138, Tongzipo Road, Yuelu District, Changsha, 410013 Hunan People’s Republic of China; 2 Department of Stomatology, The Third Xiangya Hospital of Central South University, Changsha, 410013 Hunan People’s Republic of China; 3 Department of Orthodontics, Changsha Stomatology Hospital, Changsha, 410005 Hunan People’s Republic of China; 4 Department of Spine Surgery, The Third Xiangya Hospital of Central South University, Changsha, 410013 Hunan People’s Republic of China; 5 Changsha Blood Center, Changsha, 410001 Hunan People’s Republic of China; 6 The Department of Wound Joint Surgery, Affiliated Hospital of Yiyang Medical College, Yiyang, 413000 Hunan People’s Republic of China; 7 Department of Emergency, The Third Xiangya Hospital of Central South University, Changsha, 410013 Hunan People’s Republic of China

**Keywords:** Osteoporosis, Osteoclast differentiation, CircZNF367, FUS, CRY2

## Abstract

**Background:**

Osteoporosis, characterized by reduced bone mass and deterioration of bone quality, is a significant health concern for postmenopausal women. Considering that the specific role of circRNAs in osteoporosis and osteoclast differentiation remains poorly understood, this study aims to shed light on their involvement in these processes to enhance our understanding and potentially contribute to improved treatment strategies for osteoporosis.

**Methods:**

An osteoporotic model was constructed in vivo in ovariectomized mouse. In vitro, we induced osteoclast formation in bone marrow-derived macrophages (BMDMs) using M-CSF + RANKL. To assess osteoporosis in mice, we conducted HE staining. We used MTT and TRAP staining to measure cell viability and osteoclast formation, respectively, and also evaluated their mRNA and protein expression levels. In addition, RNA pull-down, RIP and luciferase reporter assays were performed to investigate interactions, and ChIP assay was used to examine the impact of circZNF367 knockdown on the binding between FUS and CRY2.

**Results:**

We observed increased expression of CircZNF367, FUS and CRY2 in osteoporotic mice and M-CSF + RANKL-induced BMDMs. Functionally, knocking down circZNF367 inhibited osteoporosis in vivo*.* Furthermore, interference with circZNF367 suppressed osteoclast proliferation and the expression of TRAP, NFATc1, and c-FOS. Mechanistically, circZNF367 interacted with FUS to maintain CRY2 mRNA stability. Additionally, knocking down CRY2 rescued M-CSF + RANKL-induced osteoclast differentiation in BMDMs promoted by circZNF367 and FUS.

**Conclusion:**

This study reveals that the circZNF367/FUS axis may accelerate osteoclasts differentiation by upregulating CRY2 in osteoporosis and suggests that targeting circZNF367 may have potential therapeutic effects on osteoporosis.

## Introduction

Osteoporosis is a widespread condition that affects over 200 million individuals and is characterized by decreased bone mass and deterioration of bone quality [1]. Among the elderly population, osteoporosis is the main risk factor for fractures, as it may cause serious consequences for their overall quality of life and health [2]. Further, previous studies have shown that osteoporosis has a higher incidence in postmenopausal women, also known as postmenopausal osteoporosis (PMOP) [3–5]. It is well reported that osteoclasts and osteoblasts are important factors in maintaining bone homeostasis, with osteoclasts responsible for bone absorption [6]. Excessive osteoclast activation can lead to the development of osteoporosis, osteoarthritis, and rheumatoid arthritis [7, 8]. In addition, osteoporosis medications often target osteoclasts to inhibit bone resorption, underscoring the importance of investigating the regulatory network of osteoclasts for effective osteoporosis treatment.

In recent years, there has been a growing understanding of the roles played by non-coding RNAs in osteoporosis, among which one particular class of non-coding RNA, known as circular RNAs (circRNAs), has gained increasing attention. CircRNAs are primarily found in the cytoplasm of mammals and have a unique structure that confers stability and resistance to degradation by exonucleases, as they lack 5′ and 3′ ends [9]. Despite their potential importance, research on circRNAs in osteoporosis is still limited. CircRNAs often act as competitive binding agents for miRNAs, thereby modulating the inhibitory functions of miRNAs [10–12]. Several circRNAs, such as circPVT1, circ_0006873, circRNA_0001795, and circ_0005564, have been identified to regulate osteogenic differentiation by acting as sponges for specific miRNAs [10, 13–15]. Osteogenic differentiation refers to the process of precursor cells differentiating into osteoblasts (specialized cells necessary for bone structure and facilitate the deposition of calcium and phosphate, which are crucial for bone strength) for bone formation, while osteoclast differentiation involves precursor cells differentiating into osteoclasts, which are specialized cells primarily responsible for bone resorption [16, 17]. Although both osteogenic and osteoclast differentiation play crucial roles in bone biology, previous studies have primarily focused on the regulatory role of circRNAs in osteogenic differentiation [18], with limited attention given to osteoclast differentiation.

A few specific circRNAs have been implicated in the regulation of osteoclast differentiation. For instance, hsa_circ_0021739 has been found to inhibit osteoclast differentiation by reducing the levels of hsa-miR-502-5p [19]. Another circRNA, circHmbox1, exhibits reduced expression during TNF-α-induced osteoclast differentiation, suggesting its inhibitory role in this process [20]. Circ_0007059 has been shown to alleviate the inhibition of BMP2 by targeting miR-378, thereby attenuating osteoclast differentiation [21]. Additionally, circRNA_28313 has been identified as a regulator of osteoclast differentiation in primary bone marrow-derived macrophages (BMDMs) by restoring CSF1 expression by targeting mir-195a [22]. Our preliminary laboratory investigation identified circZNF367 (hsa_circ_0008842) as having protective functions in osteoporosis. Previous research has demonstrated that circZNF367 is upregulated in patients with osteoporosis, and its overexpression inhibits the migration, invasion and osteogenic differentiation of human bone marrow mesenchymal stem cells (hBMSCs) [23]. However, the precise role of circZNF367 in osteoclast differentiation remains under-investigated and unknown. Thus, by studying the role of circZNF367 in osteoclast differentiation, we can uncover its potential as a therapeutic target for osteoporosis and potentially develop novel strategies for the treatment or prevention of this debilitating bone disease.

Besides the well-known competing endogenous RNA (ceRNA) mechanism, circRNAs have also been reported to interact with RNA-binding proteins (RBPs) to exert their functions. For instance, CircStag1 has been found to promote the activity of human antigen R and activate the Wnt signaling pathway upon translocation to the cytoplasm, thereby inducing osteogenic differentiation [24]. However, no other circRNA-RBP interactions have been identified in osteoporosis thus far. Fused in sarcoma (FUS) is an important RBP involved in transcription processes [25]. It has been extensively studied in frontotemporal lobar degeneration and amyotrophic lateral sclerosis, focusing on its roles in DNA repair, transcription, dendritic RNA transport, and miRNA biogenesis [25, 26]. However, its involvement in osteoporosis remains unclear. On the other hand, cryptochrome 2 (CRY2) is a core component of clock proteins and is associated with DNA damage and repair, cell proliferation, and tumorigenesis [27, 28]. It has been implicated in the regulation of osteoporosis, with evidence suggesting its participation in osteogenic differentiation [29, 30]. Nonetheless, further investigation is needed to understand the specific function of CRY2 in osteoclast differentiation.

Our study is the first to uncover the promoting function of circZNF367 in osteoclast differentiation. Further investigations reveal that circZNF367 interacts with FUS, facilitating FUS translocation into the cytoplasm. Subsequently, FUS enhances the mRNA stability of CRY2, thereby promoting osteoclast differentiation. Inhibition of the circZNF367-FUS-CRY2 signaling pathway impeded osteoporosis and osteoclast differentiation. Collectively, these findings suggest that targeting the circZNF367-based pathway could be a promising strategy to develop more effective targeted therapies against osteoporosis.

## Methods

### Ovariectomized mouse model of osteoporosis

Eight-week-old female C57BL/6J mice (SLAC Laboratory Animal Company, China) were randomly divided into an ovariectomized (OVX) group and a sham operation (Sham) group. Animal models were established as previously described [31]. Briefly, the mice were anesthetized with an intraperitoneal injection of 2% sodium pentobarbital. A sterile median incision was made in the lower abdomen, and the abdominal muscles were separated to access the ovaries. In the OVX group, the bilateral ovaries were ligated and excised. In the Sham group, the adipose tissue surrounding the ovaries was excised instead. Penicillin was administered into the abdominal cavity, followed by suturing the incision. All mice were housed under pathogen-free conditions and provided ad libitum access to standard diet. Six weeks post-surgery, the OVX mice were further divided into three groups (n = 5/group): OVX group, OVX + sh-NC group (injected with sh-NC), and OVX + sh-circZNF367 group (injected with sh-circZNF367). The mice were maintained under standard conditions and killed after eight weeks. Femurs were collected for subsequent experiments. All animal procedures were conducted in accordance with the guidelines of the Animal Ethics Committee of The Third Xiangya Hospital, Central South University.

### Plasmid transfection

ShRNAs targeting circZNF367 (sh-circZNF367), FUS (sh-FUS) and CRY2 (sh-CRY2), as well as their corresponding negative control (sh-NC), were synthesized by GenePharma (Shanghai, China). The pcDNA3.1-circZNF367 vector (p-circZNF367) and pcDNA3.1-FUS vector (p-FUS) were also constructed by GenePharma (Shanghai, China). When the cell density of BMDMs reached approximately 80%, the cells were transfected with a mixture of plasmid DNA and Lipofectamine 3000 reagent (Invitrogen, Paisley, UK).

### TRAP staining

The tissue sections were pre-incubated in TRAP buffer at 37 °C for 40 min, then rinsed with phosphate-buffered saline (PBS) and counterstained with hematoxylin. For BMDMs, the osteoclasts were fixed with 4% paraformaldehyde and incubated in Triton X (0.5%) for 15 min. For TRAP staining, the BMDMs were incubated in TRAP solution at 37 °C for 30 min.

### Hematoxylin–eosin (HE) staining

Tibial samples were decalcified using 10% EDTA. After 21 days, the samples were embedded in paraffin. Tissue sections of 3–4 μm thickness were prepared, then subjected to dewaxing using xylene, and passed through a gradient of ethanol solutions. The sections were stained with hematoxylin for 3 min, followed by eosin staining for 2 min at room temperature. After sealing, the slides were observed and photographed using a high-resolution microscope.

### BMDMs isolation, culture and osteoclast induction

The tibiae of C57BL/6J mice were used to isolate BMDMs, which were then cultured to induce osteoclast formation. The BMDMs were incubated in a medium containing M-CSF (Peprotech, USA) for 3 days to generate pre-osteoclasts, which were then exposed to 30 ng/ml M-CSF and 50 ng/ml RANKL (Peprotech, USA) for one week to promote their maturation into mature osteoclasts [20]. After 7 days of RANKL + M-CSF treatment, differentiated osteoclasts were fixed in 4% PFA and labeled by TRAP staining.

### 3-(4,5-dimethylthiazol-2-yl)-2,5-diphenyltetrazolium bromide (MTT) assay

Transfected BMDMs were harvested and seeded into 96-well plates at a density of 3 × 10^3^ cells per well. The cells were cultured for 24 h; then, 20 μL of MTT solution (Thermo Fisher, USA) was added to each well. The plates were further incubated at 37 °C for 2 h, the supernatant was removed, and 150 μL of dimethyl sulfoxide (DMSO) was added to each well. Next, the plates were gently mixed at 25 °C for 5 min to dissolve the formazan crystals. Finally, the wells' absorbance was measured at 490 nm using a microplate reader (Thermo Fisher, USA).

### Nuclear-cytoplasmic fractionation

The BMDMs were washed three times with PBS and then suspended in a cell fraction buffer. After incubating on ice for 15 min, the BMDMs were centrifuged, and the supernatant was removed. The nuclear pellet was collected for RNA isolation using a cell disruption buffer. Finally, the extracted RNA was subjected to RT-qPCR analysis to assess gene expression levels.

### Real-time quantitative PCR (RT-qPCR)

Total RNA was isolated from tissues or BMDMs using TRIzol (Invitrogen, Carlsbad, CA, USA) according to the manufacturer's instructions. Then, complementary DNA (cDNA) was synthesized from the total RNA using reverse transcriptase (TaKaRa, Japan). qPCR was performed using SYBR Green PCR (Taraka, Japan) in ABI 7500 Instrument (Applied Biosystems, USA). The relative expression level was determined using the 2^−ΔΔCt^ method, with GAPDH used as a reference. The primer sequences are listed in Table 1.

**Table 1:** Sequence of primers for RT-qPCR

Primer	Sequence (5′ to 3′)
CircZNF367-F	TGCAGGCTCAACAGTACCAG
CircZNF367-R	CTGGGTCTTCATGGTTTGCT
CRY2-F	GGGACTCTGTCTATTGGCATCTG
CRY2-R	GTCACTCTAGCCCGCTTGGT
FUS-F	GCCAAGATCAATCCTCCATGAGTAGTG
FUS-R	TCCACGGTCCTGCTGTCCATAG
GAPDH-F	ACGGATTTGGTCGTATTGGG
GAPDH-R	TGATTTTGGAGGGATCTCGC

### Western blot

Proteins were extracted from tissues and BMDMs using the RIPA lysis buffer (Beyotime, China) supplemented with both protease inhibitor (Thermo Fisher, USA) and phosphatase inhibitor (Thermo Fisher, USA). BCA Protein Assay Kit (Beyotime, China) was used for protein quantification. Total proteins were isolated on 10% SDS-PAGE and transported to PVDF membrane (Thermo Fisher, USA). Then, the membranes were blocked with 5% non-fat milk (Merck, San Diego, USA) overnight at 4 °C and incubated with primary antibody overnight at 4 °C. After washing three times with Tris-buffered saline with Tween 20 (TBST), they were incubated with a horseradish peroxidase-conjugated secondary antibody at room temperature for 1 h. Protein bands were observed using a chemiluminescence substrate (Thermo Fisher, USA) and on the GEL-Doc 200 system (Bio-RAD, USA). The bands were analyzed using Image J (National Institutes of Health, USA). β-Actin was used as the internal reference, and the antibodies information is listed in Table 2.

**Table 2:** Antibodies

Name	Supplier	Catalog
NFATc1	Abcam	ab2796 (1:5000)
TRAP	Abcam	ab52750 (1:5000)
c-Fos	Abcam	ab222699 (1:1000)
β-actin	Abcam	ab8226 (1:5000)
Rabbit anti-mouse IgG H&L	Southern biotech	6170-05 (1: 10,000)
Goat anti-rabbit IgG H&L	Southern biotech	4050-05 (1: 20,000)

### RNA pull-down assay

For the RNA pull-down assay, biotinylated circZNF367 and CRY2 were transfected into BMDMs cells using Lipofectamine 3000 (Thermo Fisher, USA). The purified biotinylated cells were lysed and incubated with Streptavidin Agarose Beads (Life Technologies, Carlsbad, CA, USA) for 90 min at room temperature. Then, the beads were rinsed with Biotin Elution Buffer before being boiled with SDS buffer. Finally, the proteins were eluted with buffer and analyzed by performing Western blot.

### RNA immune-precipitation (RIP) assay

RIP assay was performed using an RNA-binding protein kit (Millipore, USA) and a human anti-FUS antibody. The cell extractions were incubated with magnetic beads conjugated with the anti-FUS antibody or IgG overnight at 4 °C. After the incubation, the bound RNA–protein complexes were collected, and the binding output was assessed by performing RT-qPCR.

### Fluorescence in situ hybridization (FISH) analysis

The FISH probe targeting the backsplicing junction of circZNF367 was synthesized by RiboBio (RiboBio Biotechnology, China). BMDMs were fixed with 4% paraformaldehyde for 20 min. Subsequently, proteinase K digestion was performed on the BMDMs for 30 min, following which they were subjected to dehydration using a gradient of ethanol solutions and incubated overnight at 42 °C with a hybridization reaction solution containing the FISH probe. After washing the slides thrice with PBS, the fluorescent signals were detected, and fluorescence images were captured using LSM800 confocal laser microscopy (ZEISS, Jena, Germany).

### Luciferase reporter assay

The CRY2 promoter was cloned into the PGL3-BASIC vector (Promega Corporation, USA). The BMDMs were co-transfected with plasmids containing the CRY2 promoter and either sh-NC, sh-FUS or sh-circZNF367. Renilla plasmid (Promega, USA) was used as the internal control. After 24 h, the relative luciferase activity was measured using the Dual-Luciferase Reporter Assay System (Promega, USA).

### Chromatin immunoprecipitation (ChIP)

ChIP assay was performed using a Chromatin Prep Module Kit (Thermo Fisher, USA). Cross-linked chromatin was cut into 200–1000 bp fragments using appropriate enzymes. The fragmented chromatin was then subjected to immunoprecipitation by incubating overnight at 4 °C with either Anti-FUS or Anti-IgG antibodies for full binding. Magnetic beads were added and mixed with the chromatin, followed by a 30-min reaction at 4 °C. The immunoprecipitated chromatin was subsequently detected and analyzed by RT-qPCR.

### Statistical analysis

Data analyses were performed using Prism 8.0 software (GraphPad Software, USA). The results are presented as mean ± standard deviation. The statistical significance between two groups was determined using the unpaired t-test, while comparisons among multiple groups were conducted using one-way analysis of variance (ANOVA) followed by the Newman–Keuls post hoc test. Each experiment was repeated at least three times. A *p* value less than 0.05 (*p* < 0.05) was considered statistically significant.

## Result

### Knockdown circZNF367 reduced osteoporosis in vivo

**Fig. 1:**
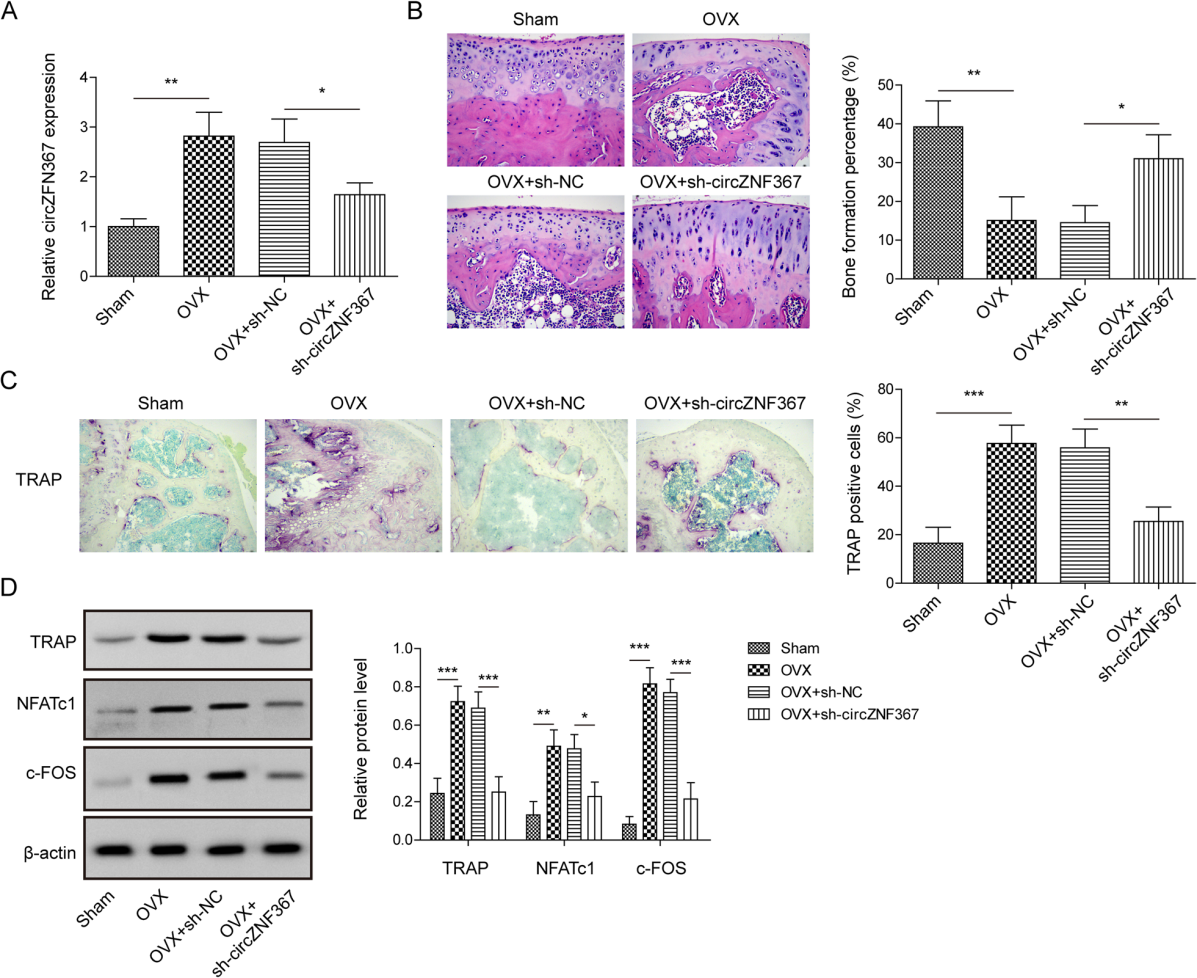
Knockdown circZNF367 reduced osteoporosis in vivo. Bilateral ovaries were surgically removed to construct the model of osteoporosis, and sh-NC or sh-circZNF367 was injected to observe the function of circZNF367 in osteoporosis. **A** Changes in mice circZNF367 expression under different treatments. **B** Representative photomicrograph of HE staining (scare bar = 50 μm). **C** TRAP staining was performed to assess osteoclast (scare bar = 50 μm). **D** Western blot was used to detect the expression of osteoclast differentiation factors in the tibia of mice. Data are represented as the means of three independent experiments ± SD. *p < 0.05, **p < 0.01, ***p < 0.001

To investigate the role of circZNF367 in osteoporosis, an osteoporosis model was established by surgically removing both ovaries in mice, while only periovarian fat was removed in the sham group. Then, shRNA targeting circZNF367 (sh-circZNF367) was injected into the tail vein to knockdown circZNF367 expression. RT-qPCR results showed a significant increase in circZNF367 expression in the osteoporotic mice, which was effectively inhibited by the tail vein injection of sh-circZNF367 (Fig. 1A). Histological examination using HE staining revealed evident characteristics of bone loss and compromised trabecular microstructure in the femur of the OVX group, along with a significant increase in the number of osteoclasts. However, inhibition of circZNF367 partially reversed these effects (Fig. 1B). The expression of NFATc1, a crucial regulator of osteoclast differentiation, was evaluated through TRAP staining, which demonstrated an increase in NFATc1-positive cells in the OVX group. However, this increase was attenuated after circZNF367 knockdown (Fig. 1C). Furthermore, the OVX group exhibited enhanced expression of key osteoclastogenic transcription factors, including c-FOS, NFATc1 and TRAP, compared to the sham group. Notably, their expression levels were partially reversed upon circZNF367 inhibition (Fig. 1D). These findings indicate that the knockdown of circZNF367 could alleviate osteoporosis in mice.

### Effects of circZNF367 knockdown upon BMDM cell osteoclast differentiation

**Fig. 2:**
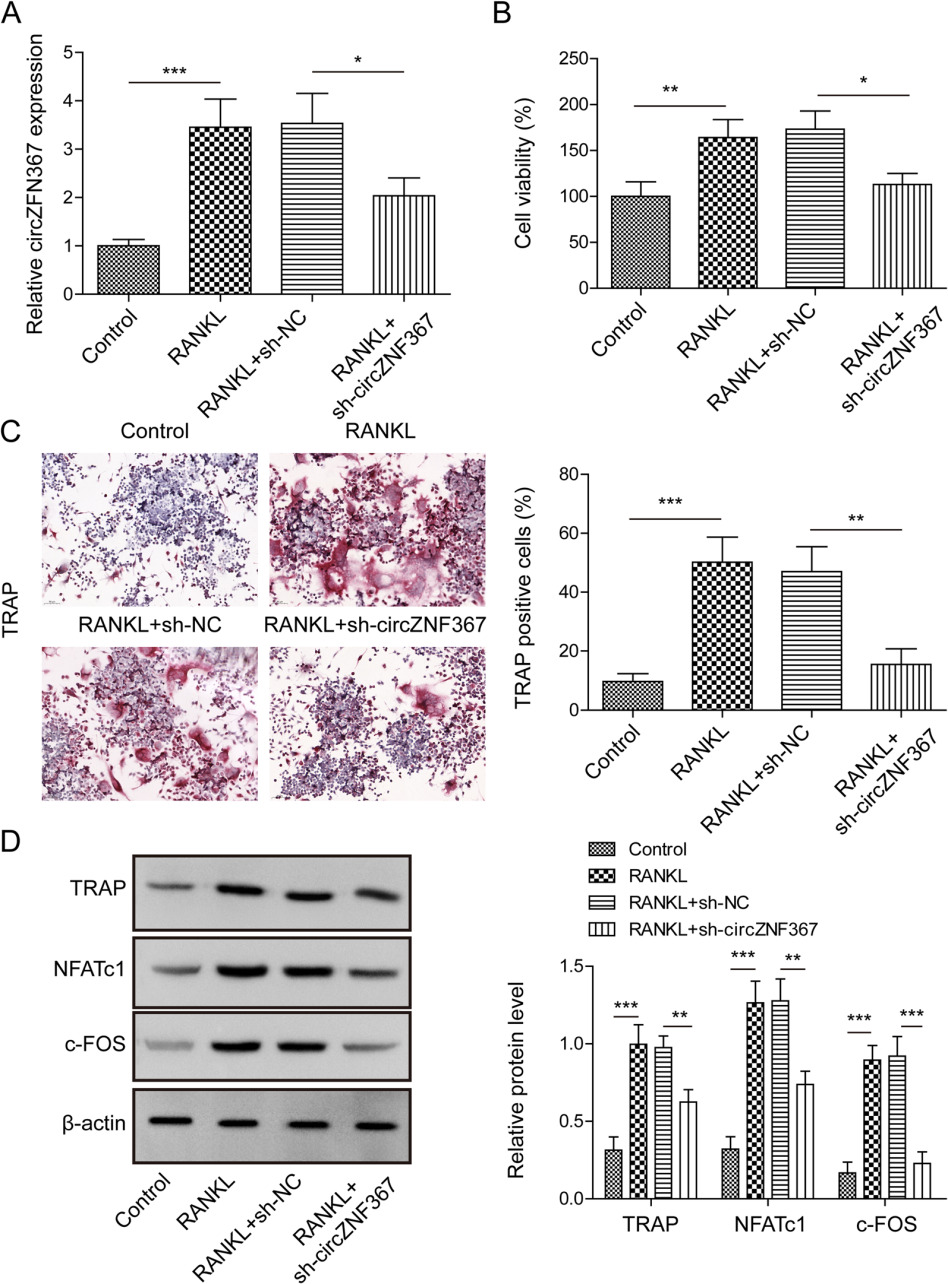
Effects of circZNF367 knockdown upon BMDMs cell osteoclast differentiation. BMDMs were harvested from mice's tibial tissues and transfected with sh-NC or sh-circZNF367. Then, BMDMs were treated with RANKL and MSF to induce osteoclast differentiation. **A** Changes in circZNF367 expression from BMDMs under different treatments. **B** Effects of circZNF367 knockdown on BMDMs proliferation. **C** TRAP staining was performed to detect osteoclast formation (scare bar = 50 μm). **D** Western blot was used to evaluate the expression of osteoclast differentiation factors in BMDMs. Data are represented as the means of three independent experiments ± SD. *p < 0.05, **p < 0.01, ***p < 0.001

In the in vitro experiments, the protective effect of circZNF367 knockdown against osteoporosis was further confirmed using RANKL and M-CSF treatment-induced osteoclast differentiation of BMDMs. The results revealed an upregulation of circZNF367 expression in RANKL-induced BMDMs (Fig. 2A) and a significant increase in osteoclast activity (Fig. 2B). However, transfection with sh-circZNF367 inhibited the proliferation of osteoclasts (Fig. 2B). TRAP staining showed an increase in osteoclasts following RANKL and M-CSF treatment, which was partially reversed by circZNF367 knockdown (Fig. 2C). Furthermore, the expression of osteoclast-associated proteins, including c-FOS, NFATc1 and TRAP, was upregulated in BMDMs induced by M-CSF and RANKL compared to the control group, and circZNF367 knockdown could partially reverse the expression levels of these proteins (Fig. 2D). Overall, the findings suggest that the knockdown of circZNF367 suppressed the differentiation of BMDMs into osteoclasts induced by M-CSF and RANKL.

### CircZNF367 interacted with FUS

**Fig. 3:**
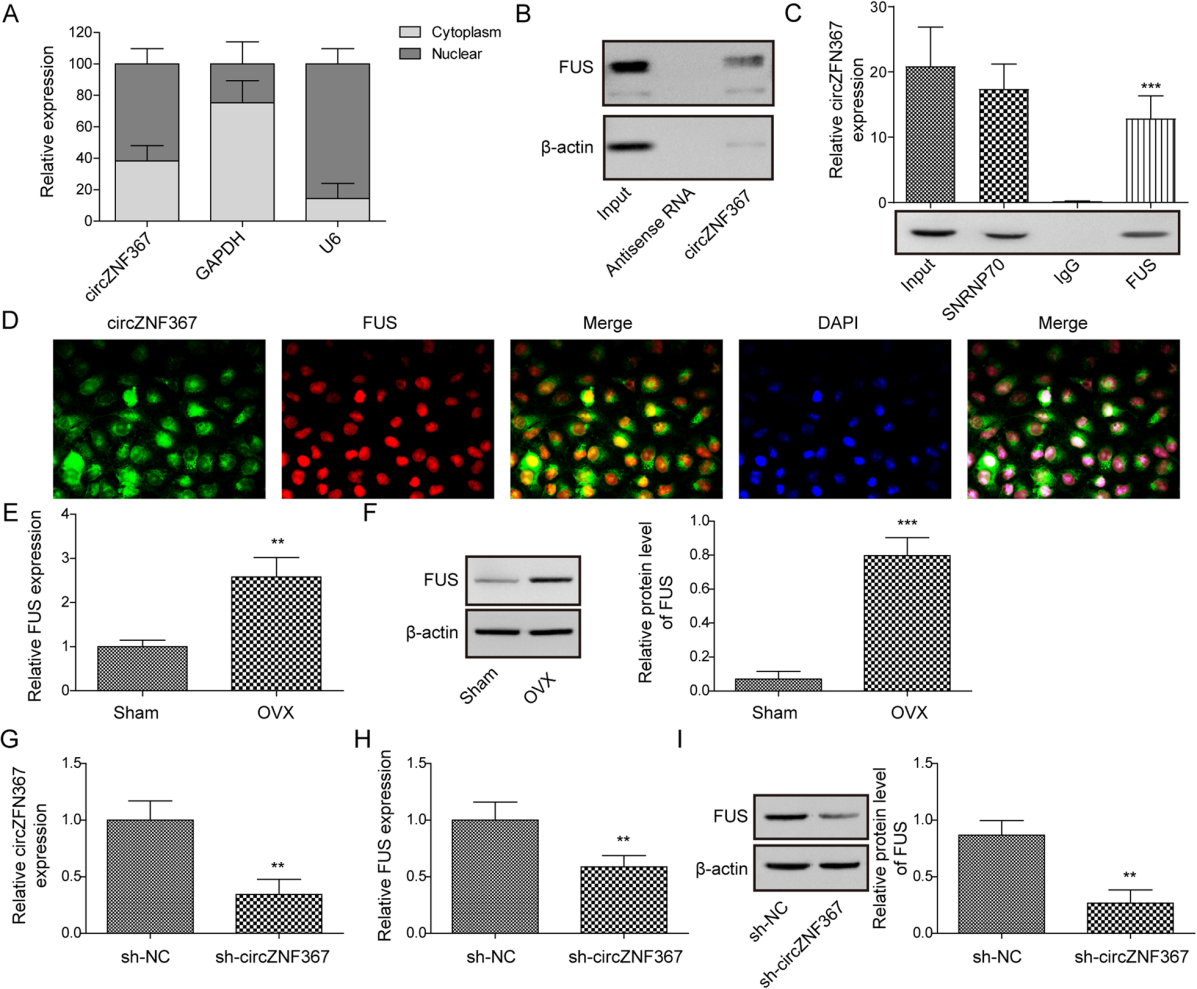
CircZNF367 interacted with FUS. **A** Expression of circZNF367 in the cytoplasm and nucleus.** B** RNA pull-down assay showing the interaction between circZNF367 and FUS. **C** The interaction between circZNF367 and FUS was determined by RIP assay. **D** FISH was conducted to detect the co-localization of circZNF367 and FUS. **E**, **F** RT-qPCR (**E**) and Western blot (**F**) validation of FUS expression in the tibia of OVA mice. **G** BMDMs were transfected with sh-NC or sh-circZNF367. Transfection efficiency was confirmed by RT-qPCR. **H**, **I** BMDMs were treated with RANKL and MSF to induce osteoclast differentiation. FUS expression in BMDMs was assessed by RT-qPCR (**H**) and Western blot (**I**). Data are represented as the means of three independent experiments ± SD. *p < 0.05, **p < 0.01, ***p < 0.001

To explore the molecular mechanism of circZNF367 regulating osteoporosis, potential targets interacting with circZNF367 were analyzed using starBase. The results of nuclear-cytoplasmic fractionation showed that circZNF367 was mainly distributed in the cytoplasm (Fig. 3A). The cell localization of circZNF367 was consistent with that of FUS, which acted as a nucleo-cytoplasmic shuttling factor regulating transcription and post-transcriptional processes. Furthermore, the interaction between circZNF367 and FUS was verified by RNA pull-down and RIP experiments (Fig. 3B, C). Additionally, the FISH assay directly demonstrated the overlapping localization of circZNF367 and FUS expression within cells (Fig. 3D). The mRNA and protein levels of FUS were found to be elevated in the OVX mice (Fig. 3E, F). Furthermore, the knockdown of circZNF367 resulted in decreased FUS expression in BMDMs (Fig. 3G, I). These findings collectively suggest that circZNF367 interacts with FUS and influences the subcellular localization of FUS proteins in BMDMs.

### CircZNF367 interacted with FUS to maintain the stability of CRY2 mRNA

**Fig. 4:**
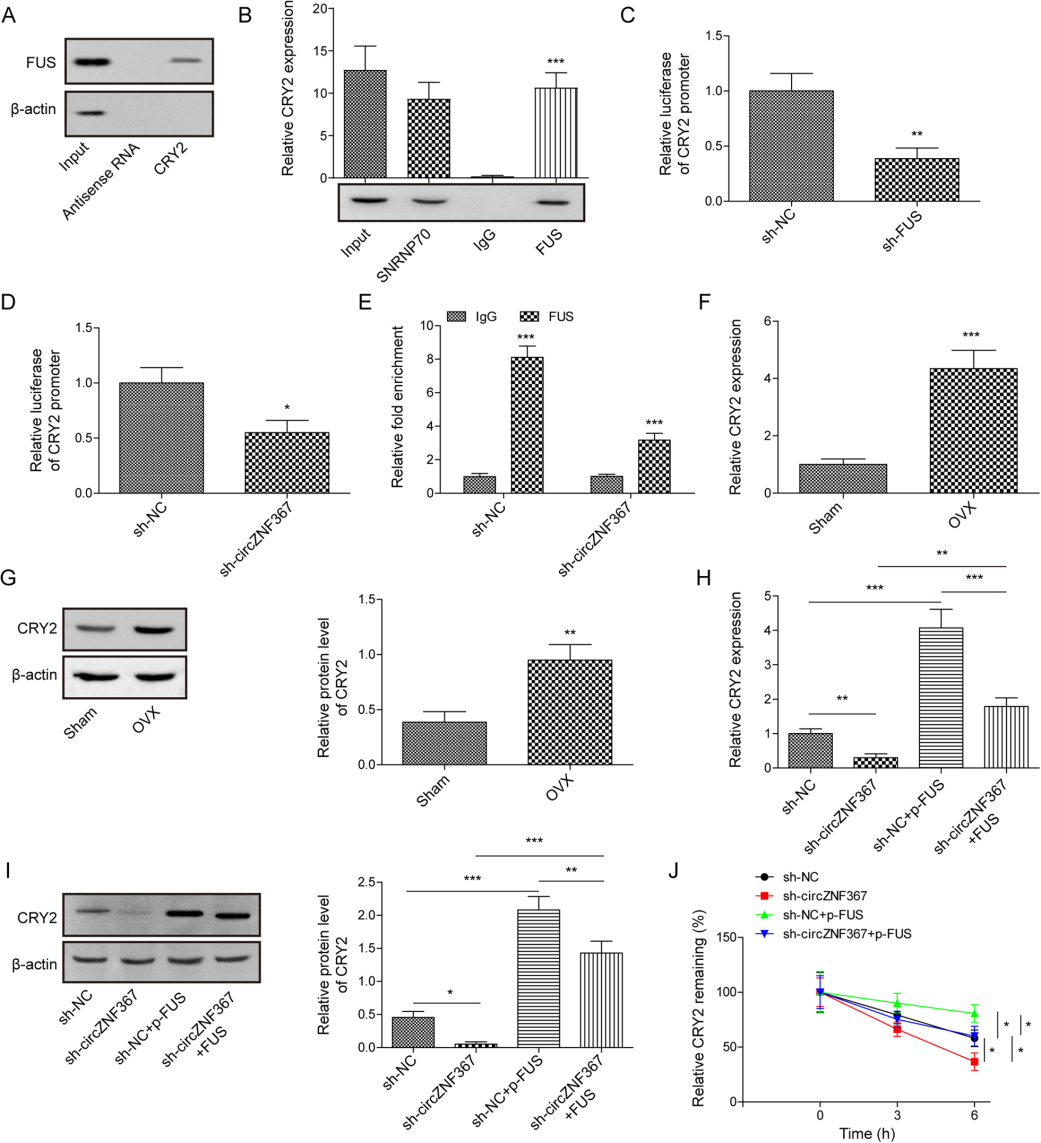
CircZNF367 interacted with FUS to maintain the stability of CRY2 mRNA. **A**, **B** RNA pull-down assay (**A**) and RIP assay (**B**) were performed to evaluate the interactions between FUS and CRY2. **C**, **D** Luciferase reporter assay was used to determine whether circZNF367 or FUS binds to CRY2. **E** The effects of circZNF367 knockdown on the binding between FUS and CRY2 were examined by ChIP assay. **F**, **G** The expression of CRY2 in OVA mice was analyzed by RT-qPCR (**F**) and Western blot (**G**). **H**, **J** BMDMs were transfected with sh-NC or sh-circZNF367 or sh-NC + pcDNA3.1-FUS or sh-circZNF367 + pcDNA3.1-FUS. The expression of CRY2 under different treatment conditions was detected by RT-qPCR (**H**) and Western blot (**I**). **J** CRY2 mRNA stability was analyzed after actinomycin D treatment. Data are represented as the means of three independent experiments ± SD. *p < 0.05, **p < 0.01, ***p < 0.001

Although it has been reported that FUS can regulate the mRNA stability of downstream target genes, the downstream molecules are not well understood [32–34]. StarBase was used to predict possible binding targets. Interestingly, the predicted results included CRY2, a regulatory factor reported to be involved in osteogenic differentiation [29]. However, the relationship between FUS and CRY2 remains unclear. The interaction between FUS and CRY2 was assessed by RNA pull-down and RIP assay (Fig. 4A, B). Furthermore, luciferase experiments demonstrated that the knockdown of FUS expression affected the CRY2 promoter activity (Fig. 4C, D). In addition, the knockdown of circZNF367 reduced CRY2 promoter activity and inhibited FUS affinity for CRY2 promoter (Fig. 4E). Further data disclosed that CRY2 mRNA expression and protein level were upregulated in OVX mice (Fig. 4F, G). As expected, sh-circZNF367 down-regulated the expression of CRY2, whereas overexpression of FUS restored CRY2 expression (Fig. 4H, I). BMDMs treated with actinomycin D showed that circZNF367 knockdown significantly down-regulated the stability of CRY2 mRNA, while FUS overexpression restored the stability of CRY2 mRNA (Fig. 4J). Altogether, these results indicate that circZNF367 could regulate CRY2 expression by targeting FUS.

### CircZNF367 and FUS promoted osteoclast differentiation of BMDMs through CRY2

**Fig. 5:**
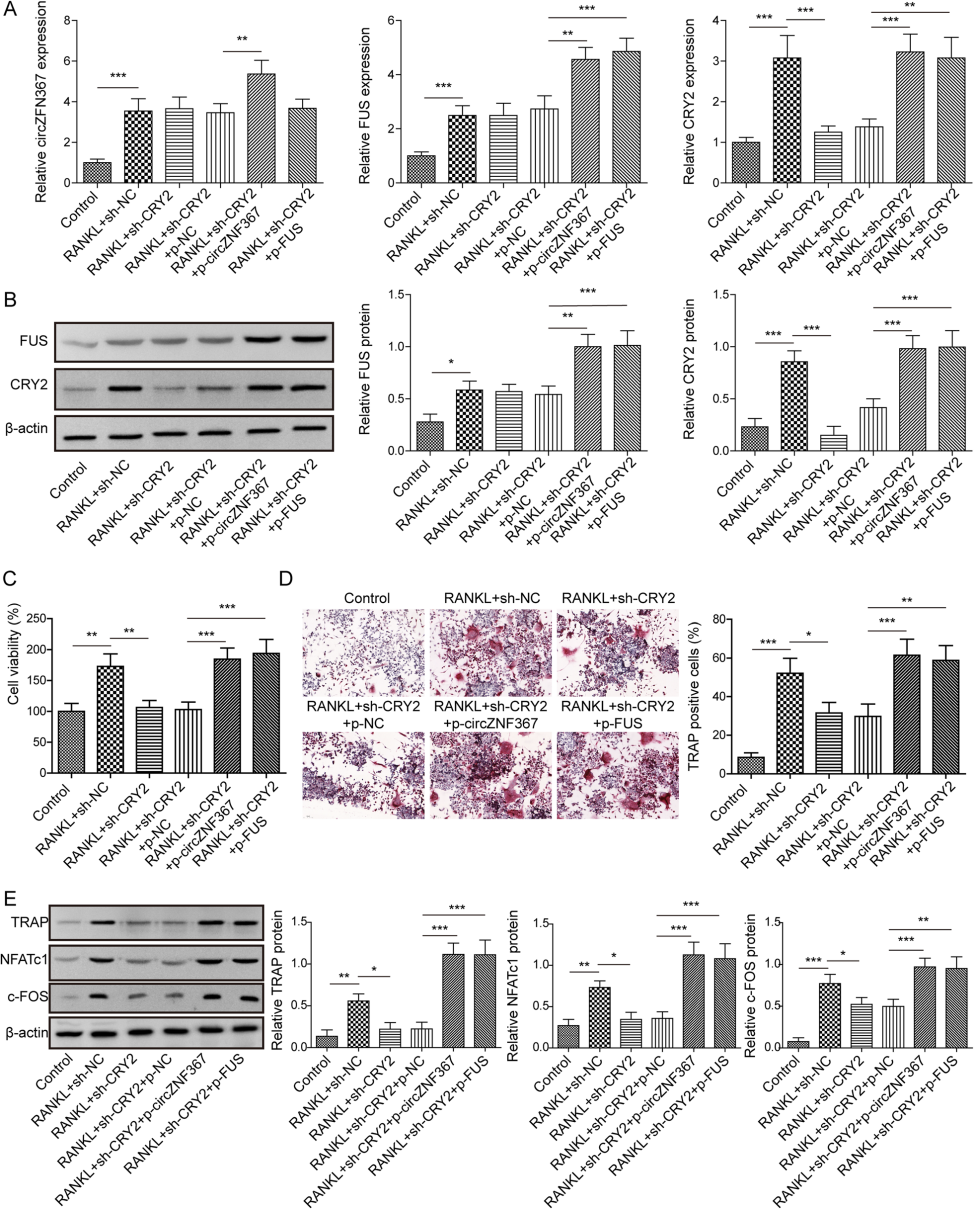
CircZNF367 and FUS promoted osteoclast differentiation of BMDMs through CRY2. BMDMs harvested from mice tibial tissues were transfected with sh-NC or sh-CRY2, or sh-CRY2 + pcDNA3.1-NC, or sh-CRY2 + pcDNA3.1-circZNF367, or sh-CRY2 + pcDNA3.1-FUS before treatment with RANKL and MSF. **A** The mRNA expression of circZNF367, FUS and CRY2 in BMDMs detected by RT-qPCR. **B** The protein levels of FUS and CRY2 in BMDMs were assessed by Western blot. **C** Cell proliferation was detected by MTT. **D** TRAP staining was used to detect osteoclast formation (scare bar = 50 μm). **E** The protein level of osteoclast differentiation factors in BMDMs was evaluated by Western blot. Data are represented as the means of three independent experiments ± SD. *p < 0.05, **p < 0.01, ***p < 0.001

To further explore the effect of the circZNF367-FUS-CRY2 signaling pathway on osteoclast differentiation, BMDMs were transfected with different plasmids and then treated with RANKL and M-CSF to induce osteoclast differentiation. The results showed that transfection of sh-CRY2 effectively attenuated CRY2 expression, while overexpression of circZNF367 or FUS restored CRY2 expression (Fig. 5A, B). Moreover, CRY2 knockdown significantly reduced BMDMs cell viability and the number of TRAP-positive cells, indicative of osteoclast differentiation. However, circZNF367 or FUS overexpression reversed the effects of CRY2 knockdown, leading to increased cell viability and the number of TRAP-positive cells (Fig. 5C, D). Furthermore, sh-CRY2 inhibited the expression of c-FOS, NFATc1 and TRAP in BMDM cells (Fig. 5E). Thus, these results confirm that CRY2 knockdown could rescue the M-CSF + RANKL-induced osteoclast differentiation of BMDMs promoted by circZNF367 and FUS.

## Discussion

Osteoporosis is a chronic bone disease that poses significant risks to the lives and well-being of the elderly, particularly postmenopausal women [35]. Disruptions in the balance between osteogenic and osteoclastic differentiation processes can contribute to the development of osteoporosis [36]. In recent years, the role of non-coding RNAs in various diseases has been elucidated. CircRNAs, characterized by their stable circular structure, have gained attention for their potential regulatory functions [37]. However, their involvement in osteoclast differentiation remains poorly understood.

This present study demonstrates that the knockdown of circZNF367 inhibits osteoclast differentiation of BMDMs and protects against osteoporosis in OVX mice. Osteoclasts are responsible for bone resorption, the process by which old or damaged bone tissue is broken down. In a healthy balance, this is followed by bone formation by osteoblasts, leading to the maintenance of bone density. However, in osteoporosis, there is a disequilibrium between bone resorption and formation, whereby excessive osteoclast differentiation and activity contribute to bone loss and compromise bone density [38]. Similarly, Liu et al. performed RNA-sequencing to identify significant differentially expressed circRNAs in osteoporosis patients and further assessed their potential functions [23]. They reported 1110 dysregulated circRNAs (474 upregulated and 636 downregulated) in the bone tissues of these patients, among which circZNF367 was among the top 10 upregulated circRNAs. Thus, this finding supports our observation of the potential beneficial effects of these findings, providing greater insights into the involvement of circZNF367 in the pathological processes leading to osteoporosis.

M-CSF and RANKL are essential factors for osteoclast differentiation and activation [39]. M-CSF promotes the proliferation, survival and differentiation of monocytes and macrophages, including osteoclast precursors, while RANKL is produced by osteoblasts and other bone cells and interacts with RANK receptors on osteoclast precursors, driving their differentiation into mature osteoclasts. When M-CSF and RANKL are combined (M-CSF + RANKL treatment), they create an environment that supports osteoclast differentiation and activation, allowing researchers to investigate the cellular and molecular mechanisms of osteoclastogenesis and bone resorption [40]. Herein, our findings reveal a significant elevation of circZNF367 in both OVX mice and M-CSF + RANKL-treated BMDMs cells, providing mechanistic insights into the role of circZNF367 in osteoclast differentiation and function. In addition, the findings that RANKL and M-CSF treatment led to an increase in TRAP-positive osteoclasts and that circZNF367 contributes to bone loss, compromised trabecular microstructure and increased osteoclasts indicate the implication of circZNF367 in the activation and differentiation of osteoclasts, which are associated with bone resorption, and that modulating circZNF367 expression or activity might potentially help reducing bone loss and the risk of fractures associated with osteoporosis [6]. The upregulation of TRAP, NFATc1 and c-FOS expression, which are well-known factors associated with osteoclast differentiation [41], suggests that monitoring changes in the expression levels of these proteins during treatment might provide insights into treatment response and allow for personalized management strategies. However, the exact mechanism by which circZNF367 regulates the expression of these factors remains unclear, and further investigation in this direction will be our next research focus.

Furthermore, we identified the circZNF367/FUS axis as a contributor to the aggravated M-CSF + RANKL-induced osteoclast differentiation in BMDMs, which could potentially occur through CRY2. FUS and CRY2 were identified as downstream targets of circZNF367, which could directly interact with FUS. circZNF367 knockdown significantly reduces FUS expression in the cytoplasm, suggesting that circZNF367 may function by recruiting FUS to the cytoplasm. FUS, a nucleus-cytoplasmic shuttling factor, can regulate the mRNA stability of downstream factors [42]. Studies have shown that FUS can bind to GluA1 to improve the mRNA stability of GluA1 and plays a role in FTLD [43]. LncXIST has been reported to regulate SPHK1 stability and downstream S1P/ERK pathway by interacting with FUS to promote osteoclast differentiation [34]. However, we are the first to reveal the regulation of FUS in osteoclast differentiation. Still, further research is required to identify and characterize additional downstream targets regulated by the circZNF367-FUS axis, which could provide further insights into the complex regulatory networks involved in osteoporosis and help the development of novel therapeutic interventions aimed at maintaining bone health through the modulations of their expression or activity.

Starbase predictions show FUS-targeted binding sites on CRY2, a transcription inhibitor, which can negatively regulate the transcription of target genes, thereby altering physiological processes in vivo [44]. It has been reported that overexpression of CRY2 reduces the osteoblastic mineralized nodules and inhibits the activation of ERK1/2, thereby inhibiting osteoblastic differentiation [30]. Another study shows that STAT3-miR-7-5p directly inhibits CRY2, activates the downstream CLOCK/BMAL1/P300 signaling pathway, and promotes osteogenic differentiation [29]. However, some researchers suggested that CRY2 deficiency in mice does not significantly affect osteoblast markers but impacts osteoclast activity [45]. Our study reveals elevated CRY2 expression in OVX mice, while inhibition of CRY2 in BMDMs significantly reduces cell viability. In addition, we determine that circZNF367 interacts with FUS to maintain CRY2 mRNA stability. The underlying mechanism of this observation remains unclear and warrants further investigation. Additionally, it is intriguing to explore how CRY2, known as a transcriptional inhibitor, could promote osteoclast differentiation and whether it has specific targets involved in this process. Therefore, further understanding the mechanisms via which CRY2 influences osteoclast differentiation and identifying potential target molecules could be important future research directions.

## Conclusion

In conclusion, our study demonstrates that circZNF367 interacts with FUS to modulate the stability of CRY2 mRNA, thereby implicating the involvement of the circZNF367-FUS-CRY2 signaling pathway in osteoporosis. These findings provide insights into the regulatory mechanisms underlying osteoporosis and suggest that targeting circZNF367 could have potential therapeutic implications for the treatment of osteoporosis.

## Data Availability

All data generated or analyzed during this study are included in this published article.
